# A Fragmentation Study on Four Oligostilbenes by Electrospray Tandem Mass Spectrometry

**DOI:** 10.1007/s13659-019-0212-3

**Published:** 2019-05-22

**Authors:** Chen-Chen Zhang, Chang-An Geng, Ji-Jun Chen

**Affiliations:** 1 State Key Laboratory of Phytochemistry and Plant Resources in West China, Kunming Institute of Botany, Chinese Academy of Sciences, 132# Lanhei Road, Kunming, 650201 Yunnan People’s Republic of China; 2 Yunnan Key Laboratory of Natural Medicinal Chemistry, Kunming, 650201 People’s Republic of China; 3 University of Chinese Academy of Sciences, Beijing, 100049 People’s Republic of China

**Keywords:** ESI-IT-TOF–MS^n^, Fragmentation rules, Oligostilbenes

## Abstract

**Abstract:**

Oligostilbenes have attracted much interest due to their intricate structures and diverse bioactivities. In this study, two stilbene dimers, (−)-7,8-*cis*-*ε*-viniferin (**1**) and carasiphenol A (**2**), and two trimers, suffruticosol A (**3**) and suffruticosol C (**4**), were investigated by electrospray ionization ion-trap time-of-flight multistage mass spectrometry (ESI-IT-TOF-MS^n^). Based on the MS^n^ study, the fragmentation pathways and diagnostic ions of four oligostilbenes in both positive and negative modes were proposed. The consecutive elimination of phenol (C_6_H_6_O) and resorcinol (C_6_H_6_O_2_) moieties were the particular dissociation for oligostilbenes due to the presence of 1,2-diphenylethylene nucleus. The present MS^n^ fragmentation study will provide valuable information for the fast characterization of oligostilbenes from complicated natural mixtures.

**Graphical Abstract:**

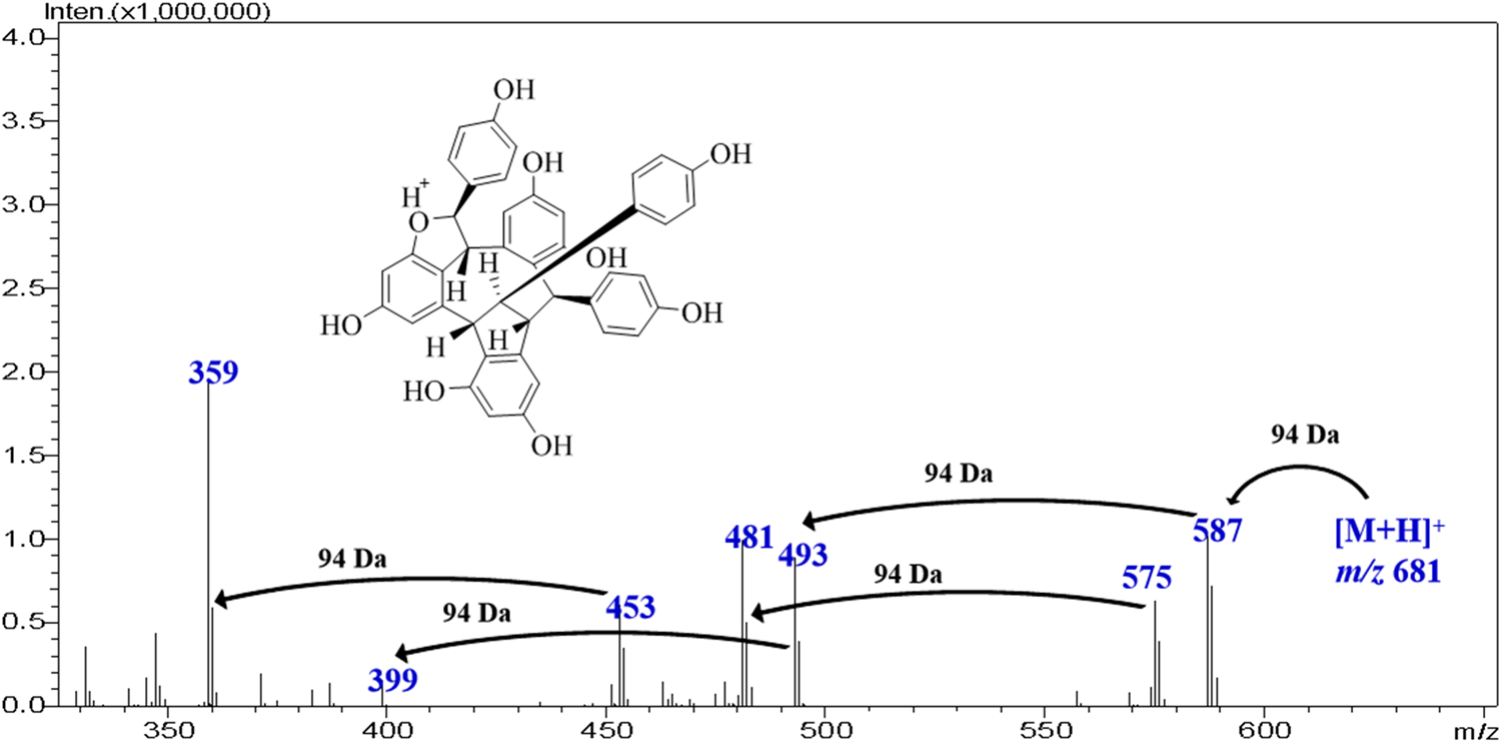

**Electronic supplementary material:**

The online version of this article (10.1007/s13659-019-0212-3) contains supplementary material, which is available to authorized users.

## Introduction

Natural stilbenes are an important group of polyphenols characterized by the presence of 1,2-diphenylethylene nucleus [1]. Naturally occurring stilbenes always have intricate structures with different numbers of stilbenes and polymeric types [2]. Stilbenes as the defensive chemicals of plants are revealed with diverse bioactivities including anti-tumor, anti-oxidant, anti-inflammatory, anti-fungal, anti-diabetic and anti-Alzheimer’s disease effects [3–7], whereas their natural distribution is quite limited, mainly in Cyperaceae, Dipterocarpaceae, Gnetaceae, Iridaceae, Leguminosae, Moraceae, Orchidaceae and Polygonaceae plants [2]. Thus, it is necessary to develop and establish a rapid and systematic method to profile stilbenes from natural resources.

Mass spectrometry (MS) with high sensitivity and resolution is one of the most efficient method in analyzing natural products [8–10]. Tandem MS techniques have advantages in ascertaining the relationship between precursor and product ions, by which the fragmentation rules and diagnostic ions of complicated compounds can be proposed [11, 12]. In this paper, we report the MS^n^ fragmentation rules of four oligostilbenes, (−)-7,8-*cis*-*ε*-viniferin (**1**), carasiphenol A (**2**), suffruticosol A (**3**) and suffruticosol C (**4**), by electrospray ionization ion-trap time-of-flight (ESI-IT-TOF) mass spectrometer to provide reference for their fast characterization from natural sources.

## Results and Discussion

**Fig. 1:**
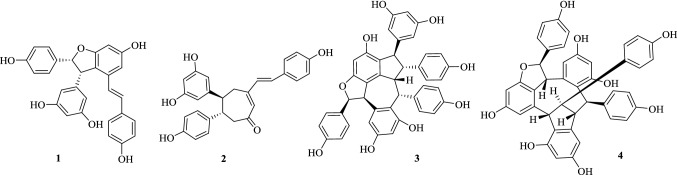
Structures of compounds **1**–**4**

**Fig. 2:**
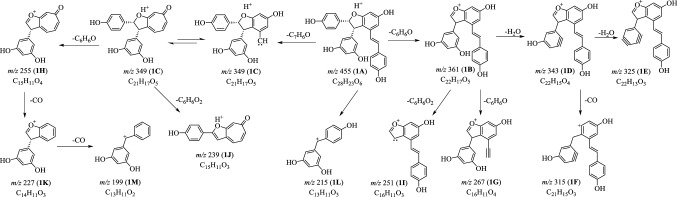
Proposed fragmentation pathways of (−)-7,8-*cis*-*ε*-viniferin (**1**) in positive mode

**Fig. 3:**
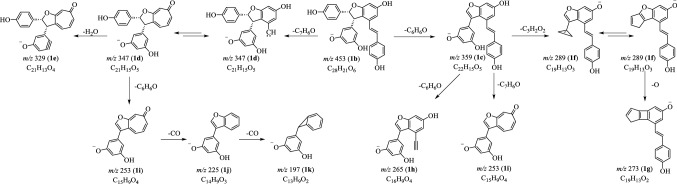
Proposed fragmentation pathways of (−)-7,8-*cis*-*ε*-viniferin (**1**) in negative mode

**Fig. 4:**
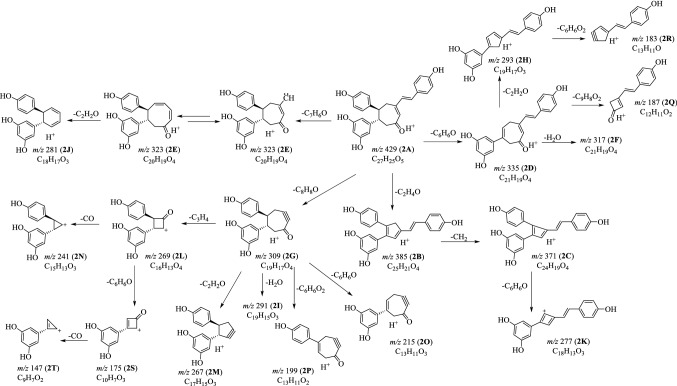
Proposed fragmentation pathways of carasiphenol A (**2**) in positive mode

**Fig. 5:**
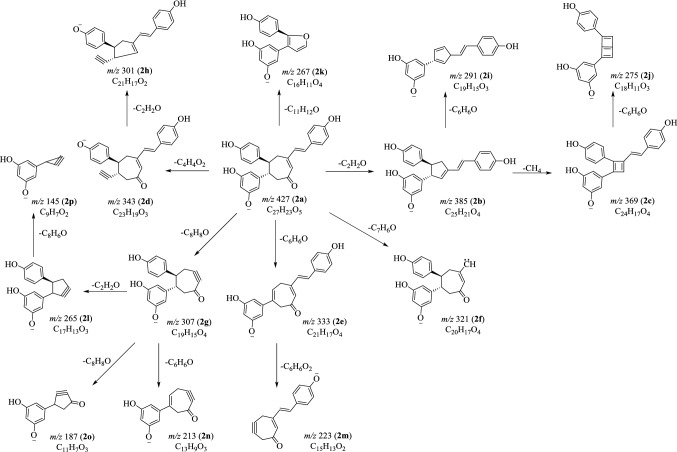
Proposed fragmentation pathways of carasiphenol A (**2**) in negative mode

**Fig. 6:**
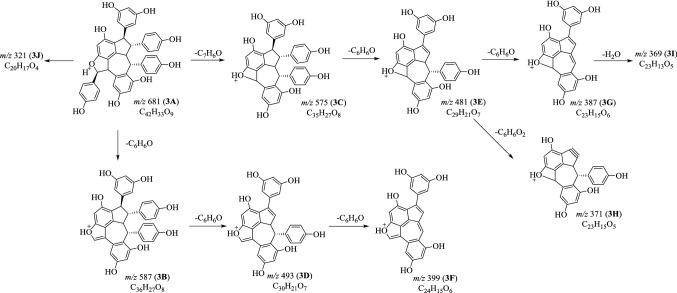
Proposed fragmentation pathways of suffruticosol A (**3**) in positive mode

**Fig. 7:**
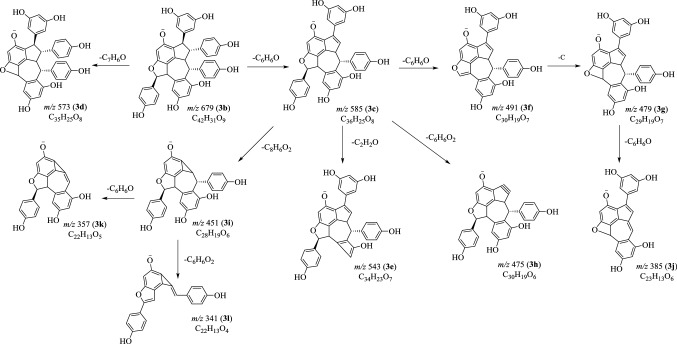
Proposed fragmentation pathways of suffruticosol A (**3**) in negative mode

**Fig. 8:**
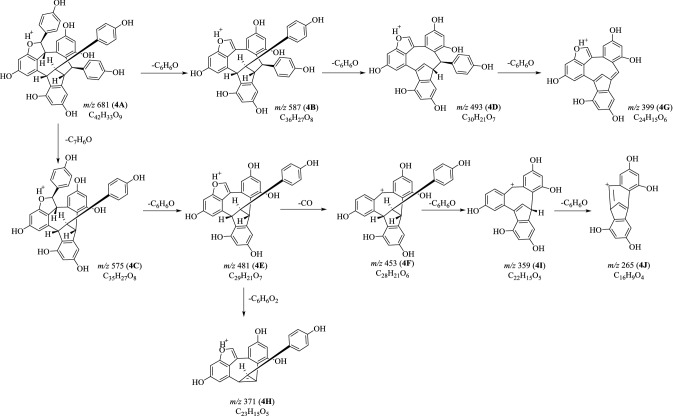
Proposed fragmentation pathways of suffruticosol C (**4**) in positive mode

**Fig. 9:**
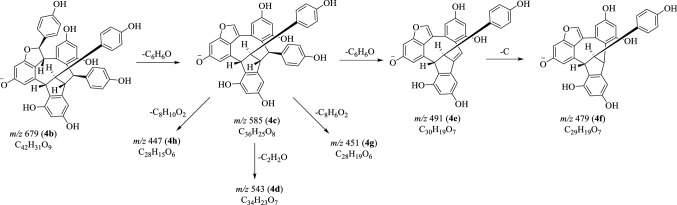
Proposed fragmentation pathways of suffruticosol C (**4**) in negative mode

The first-stage MS of compounds **1**–**4** (Fig. 1) in both positive and negative ion modes were acquired in automatic pattern, by which their protonated ([M+H]^+^) and deprotonated ([M−H]^−^) molecule ions were readily detected. For compounds **1**, **3** and **4**, the [M+HCOO]^−^ ions in negative mode were also obtained due to the application of formic acid in the solvent [13]. The subsequent MS^n^ studies on compounds **1**–**4** in both positive and negative modes were performed, from which their fragmentation pathways were proposed (Figs. 2, 3, 4, 5, 6, 7, 8, 9). It should be noted that alternative ways of fragmentation that can reasonably interpret the product ions are also possible in addition to the proposed pathway. For example, the negative charge can be present at any hydroxy group rather than the position denoted.

### MS^n^ Fragmentations of (−)-7,8-*cis*-*ε*-Viniferin (**1**) in Positive Mode

In the single-stage mass spectrum of (−)-7,8-*cis*-*ε*-viniferin (**1**), the [M+H]^+^ ion at *m*/*z* 455 (**1A**), [M−H]^−^ ion at *m*/*z* 453 (**1b**) and [M+HCOO]^−^ ion at *m*/*z* 499 (**1a**) were readily obtained, corresponding to the molecular formula of C_28_H_22_O_6_. The subsequent MS^2^ experiment on [M+H]^+^ (**1A**) gave rise to multiple product ions (**1B**–**1L**). The ion **1A** lost a C_6_H_6_O or a C_7_H_6_O moiety to provide the ions of **1B** (*m*/*z* 361) and **1C** (*m*/*z* 349) [14]. Then, the subsequent elimination of two H_2_O molecules from **1B** generated ions at *m*/*z* 343 and *m*/*z* 325. The ion **1C** was present in high abundance, which might be due to the rearrangement of **1C** into tropone derivative. The ions **1G** (*m*/*z* 267) and **1I** (*m*/*z* 251) were proposed from **1B** by the elimination of a C_6_H_6_O or a C_6_H_6_O_2_ part. The similar fragmentation was also observed for ion **1C**, from which two product ions at *m*/*z* 255 (**1H**) and *m*/*z* 239 (**1J**) were obtained corresponding to neutral loss of a C_6_H_6_O or a C_6_H_6_O_2_ part. When ion **1H** was selected as the precursor ion to perform MS^3^ experiment, two ions **1K** (*m*/*z* 227) and **1M** (*m*/*z* 199) were formed by the successive loss of two CO molecules. Similarly, the ion **1F** (*m*/*z* 315) was well explained by the loss of a molecule of CO from **1D** [15–17]. In addition, a MS^2^ ion at *m*/*z* 215 (**1L**) was also observed from **1A**, but its fragmentation pathway was still unclear (Fig. 2).

### MS^n^ Fragmentations of (−)-7,8-*cis*-*ε*-Viniferin (**1**) in Negative Mode

Similar to that in positive mode, the [M−H]^−^ ion **1b** gave rise to ions at *m*/*z* 359 (**1c**) and *m*/*z* 347 (**1d**) by the loss of a C_6_H_6_O or a C_7_H_6_O moiety. When ion **1c** was chosen to perform MS^3^ experiment, three product ions at *m*/*z* 289 (**1f**), *m*/*z* 265 (**1h**) and *m*/*z* 253 (**1i**) were obtained, corresponding to the elimination of C_3_H_2_O_2_, C_6_H_6_O and C_7_H_6_O moieties [15–17]. The MS^3^ study on **1d** provided ions **1i** (*m*/*z* 253) and **1j** (*m*/*z* 225) in accordance with the departure of C_6_H_6_O and C_7_H_6_O_2_ parts. The ions at *m*/*z* 273 (**1g**) and *m*/*z* 197 (**1k**) were tentatively deduced from **1f** and **1j** by the loss of an O or a CO moiety. Due to the presence of hydroxy groups in the structure, the elimination of H_2_O from **1d** generated **1e** (Fig. 3).

### MS^n^ Fragmentations of Carasiphenol A (**2**) in Positive Mode

The [M+H]^+^ ion **2A** gave rise to fragments at *m*/*z* 385 (**2B**), *m*/*z* 335 (**2D**) and 323 (**2E**) by the elimination of C_2_H_4_O, C_6_H_6_O and C_7_H_6_O moieties [15–17]. The ion **2E** might undergo a rearrangement and further loss a C_2_H_2_O part to provide **2J** (*m*/*z* 281). By the loss of a CH_2_ part from **2B**, the ion at *m*/*z* 371 (**2C**) was yielded, and further provided ion **2K** (*m*/*z* 277) by the loss of a C_6_H_6_O moiety. When the ion at *m*/*z* 309 (**2G**) was chosen for MS^3^ experiment, diverse ions at *m*/*z* 291 (**2I**), 267 (**2M**), 215 (**2O**) and 199 (**2P**) were obtained. The ion at *m*/*z* 269 (**2L**) was deduced from **2G** by the elimination of C_3_H_4_ part. The subsequent MS^3^ study on **2L** generated ions at *m*/*z* 241 (**2N**), 175 (**2S**) and 147 (**2T**). With the elimination of H_2_O, C_2_H_2_O or C_9_H_8_O_2_ part from **2D**, three ions at *m*/*z* 317 (**2F**), 293 (**2H**) and 187 (**2Q**) were obtained. The ion at *m*/*z* 183 (**2R**) was tentatively deduced from **2H** by the loss of resorcinol moiety (Fig. 4).

### MS^n^ Fragmentations of Carasiphenol A (**2**) in Negative Mode

The MS^2^ experiment on [M−H]^−^ ion generated prolific fragments at *m*/*z* 385 (**2b**), 369 (**2c**), 343 (**2d**), 333 (**2e**), 321 (**2f**), 307 (**2g**) and 267 (**2k**). The following MS^3^ experiment on **2b** and **2c** gave rise to **2i** (*m*/*z* 291) and **2j** (*m*/*z* 275), respectively, corresponding to the neutral loss of a phenol moiety. The ions at *m*/*z* 301 (**2h**) and 223 (**2m**) were produced from the precursors **2d** and **2e**, by the elimination of C_2_H_2_O and C_6_H_6_O_2_ [15–17]. When ion at *m*/*z* 307 (**2g**) was performed the MS^3^ study, three ions at *m*/*z* 265 (**2l**), *m*/*z* 213 (**2n**) and *m*/*z* 187 (**2o**) were formed. The ion **2p** (*m*/*z* 145) was affirmed from the precursor **2l** by the loss of a molecule of C_8_H_6_O (Fig. 5).

### MS^n^ Fragmentations of Suffruticosol A (**3**) in Positive Mode

The MS^2^ study on [M+H]^+^ ion gave rise to the fragments at *m*/*z* 587 (**3B**), 575 (**3C**), 493 (**3D**), 481 (**3E**) and 321 (**3J**). The production of ion **3E** (*m*/*z* 481) was verified as the successive elimination of a C_7_H_6_O and a C_6_H_6_O part from **3A**. The ion **3E** could further generate ion at *m*/*z* 387 (**3G**) and 371 (**3H**) by the neutral loss of a phenol (C_6_H_6_O) and a resorcinol (C_6_H_6_O_2_) moiety [15–17]. With the elimination of a molecule of H_2_O, the ion at *m*/*z* 369 (**3I**) was obtained from **3G**. Similarly, the fragment **3F** was produced from **3D** by the loss of a phenol (C_6_H_6_O) part (Fig. 6).

### MS^n^ Fragmentations of Suffruticosol A (**3**) in Negative Mode

When the [M−H]^−^ ion was chosen for MS^2^ study, the product ions at *m*/*z* 585 (**3c**) and 573 (**3d**) were generated due to the loss of C_6_H_6_O and C_7_H_6_O moieties [15–17]. The following MS^3^ investigation on ion **3c** gave rise to fragments at *m*/*z* 543 (**3e**), 491 (**3f**), 479 (**3g**) and 475 (**3h**), which could be explained by the elimination of C_2_H_2_O, C_6_H_6_O, C_7_H_6_O and C_6_H_6_O_2_. The ion **3j** (*m*/*z* 385) was deduced from **3g** by the neutral loss of a molecular of phenol (C_6_H_6_O) moiety. Two MS^3^ fragments at *m*/*z* 357 (**3k**) and 341 (**3l**) were obtained from **3i**, which were well in accordance with the departure of a phenol (C_6_H_6_O) and a resorcinol (C_6_H_6_O_2_) parts (Fig. 7).

### MS^n^ Fragmentations of Suffruticosol C (**4**) in Positive Mode

The [M+H]^+^ ion of **4A** gave rise to MS^2^ fragments at *m*/*z* 587 (**4B**) and 575 (**4C**) due to the neutral loss of a molecule of C_6_H_6_O and C_7_H_6_O parts. The successively loss of two C_6_H_6_O moieties was further observed in the MS^3^ experiment on ion **4B**, and thus gave rise to the fragments at *m*/*z* 493 (**4D**) and 399 (**4G**). The ions **4E** (*m*/*z* 481) and **4H** (*m*/*z* 371) were generated from **4C** by the successive loss of a C_6_H_6_O and a C_6_H_6_O_2_ moieties [15–17]. The ion at *m*/*z* 453 (**4F**) was deduced from **4E** by the elimination of a molecule of CO, and further gave rise to **4I** (*m*/*z* 359) and **4J** (*m*/*z* 265) which was well explained by the consecutive loss of two phenol (C_6_H_6_O) moieties. The ion at *m*/*z* 371 (**4H**) was generated from **4E** by the departure of a C_6_H_6_O_2_ moiety (Fig. 8).

### MS^n^ Fragmentations of Suffruticosol C (**4**) in Negative Mode

In the negative MS^2^ experiment, the neutral loss of C_6_H_6_O from the [M−H]^−^ ion (**4b**) gave rise to the fragment at *m*/*z* 585 (**4c**). When the ion **4c** was chosen to perform the MS^3^ study, diverse ions at *m*/*z* 543, 491, 479, 451 and 447 were obtained. The ions **4e** and **4f** were well consistent with the elimination of C_6_H_6_O and C_7_H_6_O parts from the precursor **4c** [15–17]. However, the formation of ions **4d** (*m*/*z* 543), **4g** (*m*/*z* 451) and **4h** (*m*/*z* 447) was difficult to explain due to the complicated structure (Fig. 9).

## Experimental

### Apparatus and Analytical Conditions

All of the MS^n^ experiments were performed on the LCMS-IT-TOF mass spectrometer (Shimadzu, Kyoto, Japan). Accurate masses were calibrated using sodium trifluoroacetate (CF_3_CO_2_Na) clusters. MS experiments were performed in automatic pattern, and MS^n^ experiments were achieved in direct mode. The MS parameters are in accordance with the previous report [18].

### Chemicals and Samples

Acetonitrile (CH_3_CN) of HPLC grade was purchased from Merck Co., Ltd., Germany, and formic acid was bought from Aladdin Chemistry Co., Ltd., China. Deionized water was purified using a MingChe™-D 24UV Merck Millipore system (Merck Millipore, Shanghai, China). Compounds **1**–**4** were isolated from the seeds of *Paeonia lactiflora* Pall. in our previous investigation. Samples were diluted in MeOH at the concentration of 0.5 mg/mL.

## Conclusion

The ESI multi-stage mass spectra (MS^n^) of four oligostilbenes were studied for the first time by LCMS-IT-TOF, by which their fragmentation pathways were deduced. The consecutive elimination of phenol (C_6_H_6_O) and resorcinol (C_6_H_6_O_2_) moieties from the precursor ions was the particular dissociation due to the presence of 1,2-diphenylethylene nucleus in the structure. Interestingly, the elimination of a C_7_H_6_O moiety was always detected due to the fracture of the double bond in 1,2-diphenylethylene nucleus, and this fragmentation pathway might be impelled by the rearrangement of the free radical into a stable conjugated system (e.g. tropone). Based on the fragmentation rules deduced above, (−)-7,8-*cis*-*ε*-viniferin (**1**), carasiphenol A (**2**), suffruticosol A (**3**) and suffruticosol C (**4**) could be well differentiated by their respective ion pars of 455–215, 429–267, 681–321 and 681–359 in positive mode, and 453–359, 427–307, 679–451 and 679–447 in negative mode. The present MS^n^ fragmentation study will provide valuable information for the fast characterization of oligostilbenes from complicated natural mixtures.

## Electronic supplementary material

Below is the link to the electronic supplementary material.

Supplementary material 1 (DOCX 361 kb)
