# Supplementary material for: A Fragmentation Study on Four Oligostilbenes by Electrospray Tandem Mass Spectrometry
Source: Nat Prod Bioprospect. 2019 May 22;9(4):279–86. doi: 10.1007/s13659-019-0212-3 (PMC6646503; doi:10.1007/s13659-019-0212-3)
Supplement: Supplementary file 1 — Supplementary material 1 (DOCX 361 kb) [file 13659_2019_212_MOESM1_ESM.docx]

Figure S1. Mass spectra of (‒)-7,8-*cis*-*ε*-viniferin (**1**) in positive mode

Figure S2. Mass spectra of (‒)-7,8-*cis*-*ε*-viniferin (**1**) in negative mode

Figure S3. Mass spectra of carasiphenol A (**2**) in positive mode

Figure S4. Mass spectra of carasiphenol A (**2**) in negative mode

Figure S5. Mass spectra of suffruticosol A (**3**) in positive mode

Figure S6. Mass spectra of suffruticosol A (**3**) in negative mode

Figure S7. Mass spectra of suffruticosol C (**4**) in positive mode

Figure S8. Mass spectra of suffruticosol C (**4**) in negative mode

**Table S1** Data for accurate masses and elemental composition of (‒)-7,8-*cis*-*ε*-viniferin (**1**) observed from tandem mass spectra in positive and negative modes.

| MS^n^ | Precursor  ion (*m/z*) | Product  ion (*m/z*) | Elemental  composition | Measured  (*m/z*) | Calculated  (*m/z*) | Error  (mDa) | Ion  name | Assignment |
| --- | --- | --- | --- | --- | --- | --- | --- | --- |
| (+) MS | (454) | 455 | C_28_H_23_O_6_ | 455.1490 | 455.1489 | +0.1 | **1A** | [M+H]^+^ |
| MS^2^ | 455 | 361 | C_22_H_17_O_5_ | 361.1082 | 361.1071 | +1.1 | **1B** | **1A**-C_6_H_6_O |
|  |  | 349 | C_21_H_17_O_5_ | 349.1079 | 349.1071 | +0.8 | **1C** | **1A**-C_7_H_6_O |
|  |  | 343 | C_22_H_15_O_4_ | 343.0979 | 343.0965 | +1.4 | **1D** | **1B**-H_2_O |
|  |  | 325 | C_22_H_13_O_3_ | 325.0855 | 325.0859 | -0.4 | **1E** | **1D**-H_2_O |
|  |  | 315 | C_21_H_15_O_3_ | 315.1039 | 315.1060 | -2.1 | **1F** | **1D**-CO |
|  |  | 267 | C_16_H_11_O_4_ | 267.0628 | 267.6052 | -2.4 | **1G** | **1B**-C_6_H_6_O |
|  |  | 255 | C_15_H_11_O_4_ | 255.0645 | 255.0652 | -0.7 | **1H** | **1C**-C_6_H_6_O |
|  |  | 251 | C_16_H_11_O_3_ | 251.0697 | 251.0703 | -0.6 | **1I** | **1B**-C_6_H_6_O_2_ |
|  |  | 239 | C_15_H_11_O_3_ | 239.0678 | 239.0703 | -2.5 | **1J** | **1C**-C_6_H_6_O_2_ |
|  |  | 227 | C_14_H_11_O_3_ | 227.0698 | 227.0703 | -0.5 | **1K** | **1H**-CO |
|  |  | 215 | C_13_H_11_O_3_ | 215.0688 | 215.0703 | -1.5 | **1L** |  |
| MS^3^ | 361 | 343 | C_22_H_15_O_4_ | 343.0972 | 343.0965 | +0.7 | **1D** | **1B**-H_2_O |
|  |  | 315 | C_21_H_15_O_3_ | 315.1028 | 315.1060 | -3.2 | **1F** | **1D**-CO |
|  |  | 267 | C_16_H_11_O_4_ | 267.0638 | 267.0652 | -1.4 | **1G** | **1B**-C_6_H_6_O |
|  |  | 251 | C_16_H_11_O_3_ | 251.0730 | 251.0703 | +2.7 | **1I** | **1B**-C_6_H_6_O_2_ |
|  | 349 | 255 | C_15_H_11_O_4_ | 255.0637 | 255.0652 | -1.5 | **1H** | **1C**-C_6_H_6_O |
|  |  | 239 | C_15_H_11_O_3_ | 239.0707 | 239.0703 | +0.4 | **1J** | **1C**-C_6_H_6_O_2_ |
|  | 343 | 325 | C_22_H_13_O_3_ | 325.0838 | 325.0859 | -2.1 | **1E** | **1D**-H_2_O |
|  |  | 315 | C_21_H_15_O_3_ | 315.1093 | 315.1060 | +3.3 | **1F** | **1D**-CO |
|  | 255 | 227 | C_14_H_11_O_3_ | 227.0723 | 227.0703 | +2.0 | **1K** | **1H**-CO |
|  |  | 199 | C_13_H_11_O_2_ | 199.0777 | 199.0754 | +2.3 | **1M** | **1K-CO** |
| (-) MS | (454) | 499 | C_29_H_23_O_8_ | 449.1377 | 449.1398 | -2.1 | **1a** | [M+HCOO]^-^ |
|  |  | 453 | C_28_H_21_O_6_ | 453.1329 | 453.1344 | -1.5 | **1b** | [M-H]^-^ |
| MS^2^ | 453 | 359 | C_22_H_15_O_5_ | 359.0923 | 359.0925 | -0.2 | **1c** | **1a**-C_6_H_6_O |
|  |  | 347 | C_21_H_15_O_5_ | 347.0921 | 347.0925 | -0.4 | **1d** | **1a**-C_7_H_6_O |
|  |  | 329 | C_21_H_13_O_4_ | 329.0837 | 329.0819 | +1.8 | **1e** | **1d**-H_2_O |
|  |  | 289 | C_19_H_15_O_3_ | 289.0876 | 289.0870 | +0.6 | **1f** | **1c**-C_3_H_2_O_2_ |
|  |  | 273 | C_19_H_15_O_2_ | 273.0907 | 273.0921 | -1.4 | **1g** | **1f**-O |
|  |  | 265 | C_16_H_9_O_4_ | 265.0494 | 265.0506 | -1.2 | **1h** | **1c**-C_6_H_6_O |
|  |  | 253 | C_15_H_9_O_4_ | 253.0524 | 253.0506 | +1.8 | **1i** | **1c**-C_7_H_6_O |
|  |  | 225 | C_14_H_9_O_3_ | 225.0566 | 225.0557 | +0.9 | **1j** | **1i**-CO |
|  |  | 197 | C_13_H_9_O_2_ | 197.0587 | 197.0608 | -2.1 | **1k** | **1j**-CO |
| MS^3^ | 359 | 289 | C_19_H_15_O_3_ | 289.0882 | 289.0870 | +1.2 | **1f** | **1c**-C_6_H_6_O |
|  |  | 265 | C_16_H_9_O_4_ | 265.0494 | 265.0506 | -1.2 | **1h** | **1c**-C_6_H_6_O |
|  |  | 253 | C_15_H_9_O_4_ | 253.0515 | 253.0506 | +0.9 | **1i** | **1c**-C_7_H_6_O |
|  | 347 | 253 | C_15_H_9_O_4_ | 253.0476 | 253.0506 | -3.0 | **1i** | **1c**-C_7_H_6_O |
|  |  | 225 | C_14_H_9_O_3_ | 225.0585 | 225.0557 | +2.8 | **1j** | **1i**-CO |

**Table S2** Data for accurate masses and elemental composition of carasiphenol A (**2**) observed from tandem mass spectra in positive and negative modes.

| MS^n^ | Precursor  ion (*m/z*) | Product  ion (*m/z*) | Elemental  composition | Measured  (*m/z*) | Calculated  (*m/z*) | Error  (mDa) | Ion  name | Assignment |
| --- | --- | --- | --- | --- | --- | --- | --- | --- |
| (+) MS | (428) | 429 | C_27_H_25_O_5_ | 429.1703 | 429.1697 | +0.6 | **2A** | [M+H]^+^ |
| MS^2^ | 429 | 385 | C_25_H_21_O_4_ | 385.1413 | 385.1434 | -2.1 | **2B** | **2A**-C_2_H_4_O |
|  |  | 371 | C_24_H_19_O_4_ | 371.1294 | 371.1278 | +1.6 | **2C** | **2B**-CH_2_ |
|  |  | 335 | C_21_H_19_O_4_ | 335.1285 | 335.1278 | +0.7 | **2D** | **2A**-C_6_H_6_O |
|  |  | 323 | C_20_H_19_O_4_ | 323.1297 | 323.1278 | +1.9 | **2E** | **2A**-C_7_H_6_O |
|  |  | 317 | C_21_H_17_O_3_ | 317.1185 | 317.1172 | +1.3 | **2F** | **2D**-H_2_O |
|  |  | 309 | C_19_H_17_O_4_ | 309.1128 | 309.1121 | +0.7 | **2G** | **2A**-C_8_H_8_O |
|  |  | 293 | C_19_H_17_O_3_ | 293.1165 | 293.1172 | -0.7 | **2H** | **2D**-C_2_H_2_O |
|  |  | 291 | C_19_H_15_O_3_ | 291.1000 | 291.1016 | -1.6 | **2I** | **2G**-H_2_O |
|  |  | 277 | C_18_H_13_O_3_ | 277.0863 | 277.0859 | +0.4 | **2K** | **2C**-C_6_H_6_O |
|  |  | 269 | C_16_H_13_O_4_ | 269.0813 | 269.0808 | +0.5 | **2L** | **2G**-C_3_H_4_ |
|  |  | 267 | C_17_H_15_O_3_ | 267.1010 | 267.1016 | -0.6 | **2M** | **2G**-C_2_H_2_O |
|  |  | 241 | C_15_H_13_O_3_ | 241.0850 | 241.0859 | -0.9 | **2N** | **2L**-CO |
|  |  | 215 | C_13_H_11_O_3_ | 215.0700 | 215.0703 | -0.3 | **2O** | **2G**-C_6_H_6_O |
|  |  | 199 | C_13_H_11_O_2_ | 199.0740 | 199.0754 | -1.4 | **2P** | **2G**-C_6_H_6_O_2_ |
|  |  | 187 | C_12_H_11_O_2_ | 187.0722 | 187.0754 | -3.2 | **2Q** | **2D**-C_9_H_8_O_2_ |
|  |  | 183 | C_13_H_11_O | 183.0810 | 183.0804 | +0.6 | **2R** | **2H**-C_6_H_6_O_2_ |
|  |  | 147 | C_9_H_7_O_2_ | 147.0458 | 147.0441 | +1.7 | **2T** | **2S**-CO |
| MS^3^ | 371 | 277 | C_18_H_13_O_3_ | 277.0858 | 277.0859 | -0.1 | **2K** | **2C**-C_6_H_6_O |
|  | 335 | 317 | C_21_H_17_O_3_ | 317.1185 | 317.1172 | +1.3 | **2F** | **2D**-H_2_O |
|  |  | 293 | C_19_H_17_O_3_ | 293.1165 | 293.1172 | -0.7 | **2H** | **2D**-C_2_H_2_O |
|  |  | 187 | C_12_H_11_O_2_ | 187.0722 | 187.0754 | -3.2 | **2Q** | **2D**-C_9_H_8_O_2_ |
|  | 323 | 281 | C_22_H_13_O_3_ | 281.1172 | 281.1172 | 0.0 | **2J** | **2E**-C_2_H_2_O |
|  | 309 | 291 | C_19_H_15_O_3_ | 291.1025 | 291.1016 | +0.9 | **2I** | **2G**-H_2_O |
|  |  | 267 | C_17_H_15_O_3_ | 267.1049 | 267.1016 | +3.3 | **2M** | **2G**-C_2_H_2_O |
|  |  | 215 | C_13_H_11_O_3_ | 215.0680 | 215.0703 | -2.3 | **2O** | **2G**-C_6_H_6_O |
|  |  | 199 | C_13_H_11_O_2_ | 199.0740 | 199.0754 | -1.4 | **2P** | **2G**-C_6_H_6_O_2_ |
|  | 269 | 241 | C_15_H_13_O_3_ | 241.0850 | 241.0859 | -0.9 | **2N** | **2L**-CO |
|  |  | 175 | C_10_H_7_O_3_ | 175.0420 | 175.0390 | +3.0 | **2S** | **2L**-C_6_H_6_O |
|  |  | 147 | C_9_H_7_O_2_ | 147.0422 | 147.0441 | -1.9 | **2T** | **2S**-CO |
| (-) MS | (428) | 427 | C_27_H_23_O_5_ | 427.1531 | 427.1551 | -2.0 | **2a** | [M-H]^-^ |
| MS^2^ | 427 | 385 | C_25_H_21_O_4_ | 385.1430 | 385.1445 | -1.5 | **2b** | **2a**-C_2_H_2_O |
|  |  | 369 | C_24_H_17_O_4_ | 369.1134 | 369.1132 | +0.2 | **2c** | **2b**-CH_4_ |
|  |  | 343 | C_23_H_19_O_3_ | 343.1327 | 343.1340 | -1.3 | **2d** | **2a**-C_4_H_4_O_2_ |
|  |  | 333 | C_21_H_17_O_4_ | 333.1139 | 333.1132 | +0.7 | **2e** | **2a**-C_6_H_6_O |
|  |  | 321 | C_20_H_17_O_4_ | 321.1118 | 321.1132 | -1.4 | **2f** | **2a**-C_7_H_6_O |
|  |  | 307 | C_19_H_15_O_4_ | 307.0987 | 307.0976 | +1.1 | **2g** | **2a**-C_8_H_8_O |
|  |  | 267 | C_16_H_11_O_4_ | 267.0676 | 267.0663 | +1.3 | **2k** | **2a**-C_11_H_12_O |
|  |  | 265 | C_17_H_13_O_3_ | 265.0885 | 265.0870 | +1.5 | **2l** | **2g**-C_2_H_2_O |
|  |  | 223 | C_15_H_13_O_2_ | 223.0787 | 223.0765 | +2.2 | **2m** | **2e**-C_6_H_6_O_2_ |
|  |  | 213 | C_13_H_9_O_3_ | 213.0581 | 213.0557 | +2.4 | **2n** | **2g**-C_6_H_6_O |
| MS^3^ | 385 | 291 | C_19_H_15_O_3_ | 291.0849 | 291.0874 | -2.5 | **2i** | **2b**-C_6_H_6_O |
|  | 369 | 275 | C_18_H_11_O_3_ | 275.0710 | 275.0714 | -0.4 | **2j** | **2c**-C_6_H_6_O |
|  | 343 | 301 | C_21_H_17_O_2_ | 301.1206 | 301.1234 | -2.8 | **2h** | **2d**-C_2_H_2_O |
|  | 333 | 223 | C_15_H_13_O_2_ | 223.0798 | 223.0765 | +3.3 | **2m** | **2e**-C_6_H_6_O_2_ |
|  | 307 | 265 | C_17_H_13_O_3_ | 265.0874 | 265.0870 | +0.4 | **2l** | **2g**-C_2_H_2_O |
|  |  | 213 | C_13_H_9_O_3_ | 213.0581 | 213.0557 | +2.4 | **2n** | **2g**-C_6_H_6_O |
|  |  | 187 | C_11_H_7_O_3_ | 187.0386 | 187.0401 | +1.5 | **2o** | **2g**-C_8_H_8_O |
|  | 265 | 145 | C_9_H_7_O_2_ | 145.0330 | 145.0295 | +3.5 | **2p** | **2l**-C_8_H_6_O |

**Table S3** Data for accurate masses and elemental composition of suffruticosol A (**3**) observed from tandem mass spectra in positive and negative modes.

| MS^n^ | Precursor  ion (*m/z*) | Product  ion (*m/z*) | Elemental  composition | Measured  (*m/z*) | Calculated  (*m/z*) | Error  (mDa) | Ion  name | Assignment |
| --- | --- | --- | --- | --- | --- | --- | --- | --- |
| (+) MS | (680) | 681 | C_42_H_33_O_9_ | 680.2091 | 680.2119 | -2.8 | **3A** | [M+H]^+^ |
| MS^2^ | 681 | 587 | C_36_H_27_O_8_ | 587.1682 | 587.1700 | -1.8 | **3B** | **3A**-C_6_H_6_O |
|  |  | 575 | C_35_H_27_O_8_ | 575.1705 | 575.1700 | +0.5 | **3C** | **3A**-C_7_H_6_O |
|  |  | 493 | C_30_H_21_O_7_ | 493.1301 | 493.1282 | +1.9 | **3D** | **3B**-C_6_H_6_O |
|  |  | 481 | C_29_H_21_O_7_ | 481.1309 | 481.1282 | +2.7 | **3E** | **3C**-C_6_H_6_O |
|  |  | 321 | C_20_H_17_O_4_ | 321.1147 | 321.1121 | +2.6 | **3J** | **3B**-C_16_H_10_O_4_ |
| MS3 | 575 | 481 | C_29_H_21_O_7_ | 481.1283 | 481.1282 | +0.1 | **3E** | **3C**-C_6_H_6_O |
|  |  | 387 | C_23_H_15_O_6_ | 387.0887 | 387.0863 | +2.4 | **3G** | **3E**-C_6_H_6_O |
|  |  | 371 | C_23_H_15_O_5_ | 371.0942 | 371.0914 | +2.8 | **3H** | **3E**-C_6_H_6_O_2_ |
|  | 493 | 399 | C_24_H_15_O_6_ | 399.0850 | 399.0863 | -1.3 | **3F** | **3D**-C_6_H_6_O |
|  | 481 | 387 | C_23_H_15_O_6_ | 387.0874 | 387.0863 | +1.1 | **3G** | **3E**-C_6_H_6_O |
|  |  | 371 | C_23_H_15_O_5_ | 371.0886 | 371.0914 | -2.8 | **3H** | **3E**-C_6_H_6_O_2_ |
| MS^4^ | 387 | 369 | C_23_H_13_O_5_ | 369.0772 | 369.0758 | +1.4 | **3I** | **3G**-H_2_O |
| (-) MS | (680) | 725 | C_43_H_33_O_11_ | 725.2011 | 725.2028 | -1.7 | **3a** | [M+HCOO]^-^ |
|  |  | 679 | C_42_H_31_O_9_ | 679.1976 | 679.1974 | +0.2 | **3b** | [M-H]^-^ |
| MS^2^ | 679 | 585 | C_36_H_25_O_8_ | 585.1541 | 585.1555 | -1.4 | **3c** | **3b**-C_6_H_6_O |
|  |  | 573 | C_35_H_25_O_8_ | 573.1554 | 573.1555 | -0.1 | **3d** | **3b**-C_7_H_6_O |
|  |  | 543 | C_34_H_23_O_7_ | 543.1451 | 543.1449 | +0.2 | **3e** | **3c**-C_2_H_2_O |
|  |  | 491 | C_30_H_19_O_7_ | 491.1137 | 491.1136 | +0.1 | **3f** | **3c**-C_6_H_6_O |
|  |  | 479 | C_29_H_19_O_7_ | 479.1135 | 479.1136 | -0.1 | **3g** | **3c**-C_7_H_6_O |
|  |  | 475 | C_30_H_19_O_7_ | 475.1193 | 475.1187 | +0.6 | **3h** | **3c**-C_6_H_6_O_2_ |
|  |  | 451 | C_28_H_19_O_6_ | 451.1210 | 451.1187 | +2.3 | **3i** | **3c**-C_8_H_6_O_2_ |
|  |  | 385 | C_23_H_13_O_6_ | 385.0742 | 385.0718 | +2.4 | **3j** | **3c**-C_13_H_12_O_2_ |
| MS^3^ | 585 | 543 | C_34_H_23_O_7_ | 543.1419 | 543.1449 | -3.0 | **3e** | **3c**-C_2_H_2_O |
|  |  | 491 | C_30_H_19_O_7_ | 491.1122 | 491.1136 | -1.4 | **3f** | **3c**-C_6_H_6_O |
|  |  | 479 | C_29_H_19_O_7_ | 479.1116 | 479.1136 | -2.0 | **3g** | **3c**-C_7_H_6_O |
|  |  | 475 | C_30_H_19_O_7_ | 475.1205 | 475.1187 | +1.8 | **3h** | **3c**-C_6_H_6_O_2_ |
|  |  | 451 | C_28_H_19_O_6_ | 451.1188 | 451.1187 | +0.1 | **3i** | **3c**-C_8_H_6_O_2_ |
|  |  | 385 | C_23_H_13_O_6_ | 385.0700 | 385.0718 | -1.8 | **3j** | **3c**-C_13_H_12_O_2_ |
|  | 451 | 357 | C_22_H_13_O_5_ | 357.0711 | 357.0768 | -5.7 | **3k** | **3i**-C_6_H_6_O |
|  |  | 341 | C_22_H_13_O_4_ | 341.0830 | 341.0819 | +1.1 | **3l** | **3i**-C_6_H_6_O_2_ |

**Table S4** Data for accurate masses and elemental composition of suffruticosol C (**4**) observed from tandem mass spectra in positive and negative modes.

| MS^n^ | Precursor  ion (*m/z*) | Product  ion (*m/z*) | Elemental  composition | Measured  (*m/z*) | Calculated  (*m/z*) | Error  (mDa) | Ion  name | Assignment |
| --- | --- | --- | --- | --- | --- | --- | --- | --- |
| (+) MS | (680) | 681 | C_42_H_33_O_9_ | 681.2120 | 681.2119 | +0.1 | **4A** | [M+H]^+^ |
| MS^2^ | 681 | 587 | C_36_H_27_O_8_ | 587.1673 | 587.1700 | -2.7 | **4B** | **4A**-C_6_H_6_O |
|  |  | 575 | C_35_H_27_O_8_ | 575.1691 | 575.1700 | -0.9 | **4C** | **4A**-C_7_H_6_O |
|  |  | 493 | C_30_H_21_O_7_ | 493.1281 | 493.1282 | -0.1 | **4D** | **4B**-C_6_H_6_O |
|  |  | 481 | C_29_H_21_O_7_ | 481.1291 | 481.1282 | +0.9 | **4E** | **4C**-C_6_H_6_O |
|  |  | 453 | C_28_H_21_O_6_ | 453.1361 | 453.1333 | +2.8 | **4F** | **4A**-C_14_H_12_O_3_ |
|  |  | 399 | C_24_H_15_O_6_ | 399.0880 | 399.0863 | +1.7 | **4G** | **4D**-C_6_H_6_O |
|  |  | 371 | C_16_H_9_O_4_ | 371.0947 | 371.0914 | +3.3 | **4H** | **4E**-C_6_H_6_O_2_ |
|  |  | 359 | C_22_H_15_O_5_ | 359.0936 | 359.0914 | +2.2 | **4I** | **4F**-C_6_H_6_O |
|  |  | 265 | C_16_H_9_O_4_ | 265.0515 | 265.0495 | +2.0 | **4J** | **4I**-C_6_H_6_O |
| MS^3^ | 587 | 493 | C_30_H_21_O_7_ | 493.1288 | 493.1282 | +0.6 | **4D** | **4B**-C_6_H_6_O |
|  |  | 399 | C_24_H_15_O_6_ | 399.0868 | 399.0863 | +0.5 | **4G** | **4D**-C_6_H_6_O |
|  | 575 | 481 | C_29_H_21_O_7_ | 481.1286 | 481.1282 | +0.4 | **4E** | **4C**-C_6_H_6_O |
|  |  | 371 | C_16_H_9_O_4_ | 371.0937 | 371.0914 | +2.3 | **4H** | **4E**-C_6_H_6_O_2_ |
|  | 493 | 399 | C_24_H_15_O_6_ | 399.0867 | 399.0863 | +0.4 | **4G** | **4D**-C_6_H_6_O |
|  | 453 | 359 | C_22_H_15_O_5_ | 359.0908 | 359.0914 | -0.6 | **4I** | **4F**-C_6_H_6_O |
| MS^4^ | 359 | 265 | C_16_H_9_O_4_ | 265.0481 | 265.0495 | -1.4 | **4J** | **4I**-C_6_H_6_O |
| (-) MS | (680) | 725 | C_43_H_33_O_11_ | 725.2013 | 725.2028 | -1.5 | **4a** | [M+HCOO]^-^ |
|  |  | 679 | C_42_H_31_O_9_ | 679.1976 | 679.1974 | +0.2 | **4b** | [M-H]^-^ |
| MS^2^ | 679 | 585 | C_36_H_25_O_8_ | 585.1538 | 585.1555 | -1.7 | **4c** | **4b**-C_6_H_6_O |
|  |  | 543 | C_34_H_23_O_7_ | 543.1448 | 543.1449 | -0.1 | **4d** | **4c**-CO |
|  |  | 491 | C_30_H_19_O_7_ | 491.1114 | 491.1136 | -2.2 | **4e** | **4c**-C_6_H_6_O |
|  |  | 479 | C_29_H_19_O_7_ | 479.1152 | 479.1136 | +1.6 | **4f** | **4e**-C |
|  |  | 451 | C_28_H_19_O_6_ | 451.1211 | 451.1187 | +2.4 | **4g** | **4c**-C_8_H_6_O_2_ |
|  |  | 447 | C_28_H_15_O_6_ | 447.0893 | 447.0874 | +1.9 | **4h** | **4c**-C_8_H_10_O_2_ |
| MS^3^ | 585 | 543 | C_34_H_23_O_7_ | 543.1423 | 543.1449 | -2.6 | **4d** | **4c**-CO |
|  |  | 491 | C_30_H_19_O_7_ | 491.1109 | 491.1136 | -2.7 | **4e** | **4c**-C_6_H_6_O |
|  |  | 479 | C_29_H_19_O_7_ | 479.1120 | 479.1136 | -1.6 | **4f** | **4e**-C |
|  |  | 451 | C_28_H_19_O_6_ | 451.1183 | 451.1187 | -0.4 | **4g** | **4c**-C_8_H_6_O_2_ |
|  |  | 447 | C_28_H_15_O_6_ | 447.0879 | 447.0874 | +0.5 | **4h** | **4c**-C_8_H_10_O_2_ |
